# Down regulating PHGDH affects the lactate production of sertoli cells in varicocele

**DOI:** 10.1186/s12958-020-00625-9

**Published:** 2020-07-14

**Authors:** Wen-bin Guo, Zhen-hui Huang, Cheng Yang, Xian-yuan Lv, Hui Xia, Hu Tian, Jian-kun Yang, Qi-zhao Zhou, Ming-kun Chen, Kang-yi Xue, Cun-dong Liu

**Affiliations:** Department of Urology, the Third Affiliated Hospital of Southern Medical University, Guangzhou, China

**Keywords:** Varicocele, Phosphoglycerate dehydrogenase (PHGDH), Glycolysis, Lactate metabolism, Sertoli cells (SC)

## Abstract

**Background:**

Although varicocele is considered to be one of the leading causes of male infertility, the precise mechanism underlying how varicocele leads to male infertility is not completely understood. We found the lactate concentration on the varicocele side of the patients was decreased compare with peripheral venous blood. In the testicles, the lactate produced by the sertoli cells through the glycolysis pathway provides most of the energy needed for spermatogenesis, the reduction of lactate will affect spermatogenesis. The objective of this study was to investigate the mechanism of this abnormal energy metabolism phenomenon in varicocele.

**Methods:**

In this study, we collected the testicular tissue from patients with varicocele, the glycolysis related proteins PHGDH was identified by iTRAQ proteomics technology. Experimental rat varicocele model was constructed according to our new clip technique, the mRNA and protein expression levels of PHGDH were examined with qRT-PCR and Western blotting. We constructed a sertoli cell of PHGDH down-regulation model, and then detected the glucose consumption, LDH activities and lactate production in the sertoli cells. Western blot was conducted to investigate the effects of PHGDH on the expression of phosphoserine phosphatase (PSPH) and Pyruvate kinase M2 (PKM2). Flow cytometry was used to detect the cell apoptosis and cell cycle in sertoli cells.

**Results:**

The results showed that testicular protein PHGDH was down-regulated in patients with varicocele and in experimental rat varicocele model. Down-regulation of PHGDH in sertoli cells significantly decreased the glucose consumption, LDH activities and lactate production in the sertoli cells, indicating that the low expression of PHGDH ultimately led to a decrease in lactate production by affecting the glycolysis. The Western blot results showed that the down-regulation of PHGDH significantly reduced the expression of pathway protein PSPH and PKM2, leading to the reduction of lactate production. Moreover, PHGDH knockdown can promote apoptosis and inhibit cell cycle to affect cell growth.

**Conclusions:**

Overall, we conformed that varicocele lead to the decreasing of testis lactate production. Down-regulation of PHGDH in sertoli cells may mediate the process of abnormal glucose metabolism. Our study provide new insight into the mechanisms underlying metabolism-associated male infertility and suggests a novel therapeutic target for male infertility.

## Background

Varicocele, the abnormal dilation and tortuosity of the pampiniform venous plexus within the spermatic cord, is present in about 15% of the male adult population and almost 40% of infertile males [1]. Many factors account for male infertility in varicocele, such as hypoxia, metabolic abnormalities, hormonal dysfunction, elevated testicular temperature and spermatic veins hypertension [2]. The precise mechanism by which varicocele might cause infertility is still unknown. No single factor is believed to be responsible for the negative testicular effects [3]; instead the pathogenesis is believed to be complex and multifactorial, with several proposed mechanisms acting together [4]. In this complex pathophysiological network, metabolic abnormalities seems to have a important role, although some studies have suggested a link between abnormal glycolysis and varicocele, the specific mechanisms are still poorly understood [5].

Sertoli cells, located in the basal compartment of seminiferous tubules, often referred to as nurse cells, are responsible for providing energy and nutritional support to developing germ cells [6]. It is imperative that germ cells receive an adequate level of energy substrates, otherwise they will degenerate and enter the apoptotic pathway [7]. In fact, the majority of energy required for spermatogenesis are provided by sertoli cells through glycolysis to produce lactate [8]. Therefore, the glycolysis of sertoli cells defines the population size of germ cells, which is essential for the maintenance of spermatogenesis and consequently, male fertility [9].

PHGDH, the first enzyme branching from glycolysis in a three-step serine biosynthetic pathway uses NAD+ as a cofactor to oxidize 3-phosphoglycerate into phosphohydroxypyruvate. The product is then subsequently converted into phosphoserine via transamination by PSAT1 and, ultimately, to serine via phosphate ester hydrolysis and the enzyme PSPH [10], serine is also an activator of PKM2 in the glycolysis [11]. The overexpression of PHGDH is often associated with progression of cancers, and the inhibitors of PHGDH reduce the glycolysis and suppress the growth of cancers [12]. However, the associated investigation in the reproductive system remains limited.

In this study, We found for the first time that the lactate on the varicocele side of the patients decreased and testicular protein PHGDH was down-regulated in varicocele. We revealed that varicocele lead to the low expression of PHGDH in sertoli cells and the low expression of PHGDH ultimately led to a decrease in lactate production by affecting the glycolysis pathway. Moreover, PHGDH knockdown can promote apoptosis and inhibit cell cycle to affect cell growth. That may eventually lead to impairment of spermatogenesis and male infertility. This may help to better understand the major proteins contributing to male infertility in varicocele, further exploring the mechanisms underlying male infertility, and developing novel treatments for varicocele.

## Methods

### Blood gas analysis in patients with varicocele

During laparoscopic varicocelectomy, after exposing the testicular vein, Venous blood is extracted with a syringe with a slender needle before high ligation, and the scrotum is squeezed as the blood is extracted. Finally, blood gas analysis was performed on the varicocele side venous blood and peripheral venous blood of patients at the same time to detect lactate concentration. This study was approved by the ethics committee of the Third Affiliated Hospital of Southern Medical University under code number 68222115.

### The establishment and assessment of the experimental rat varicocele model

Twenty adult male specific-pathogen free Sprague-Dawley rats (body weight, 250–300 g) were randomly assigned to a sham group (*n* = 10) and a varicocele group (n = 10). Rats were housed in a climate-controlled environment with free access to food and water under a 12-h day/night cycle. Experimental rat varicocele model was constructed according to our new clip technique [13]. The establishment and assessment of the rat model is described in the previous articles published by our team. This study was approved by the ethics committee of the Third Affiliated Hospital of Southern Medical University and the animal work was approved by the Animal Care and Ethics Committee of Southern Medical University.

### RNA isolation and qRT PCR analysis

Total RNA was extracted from the left testis of each animal using TRIzol reagent (Invitrogen, Carlsbad, USA) in accordance with the manufacturer’s instructions. In brief, tissues were homogenized and incubated in TRIzol for 10 min, and then RNA was separated using chloroform (0.2 mL/1 mL TRIzol). After centrifugation at 12,000 *g* for 15 min at 4 °C, the aqueous phase of the sample was transferred to a fresh tube for RNA precipitation using isopropyl alcohol (0.5 mL/1 mL TRIzol). After repeating the incubation and centrifugation steps, the remaining pellet was washed with 75% ethanol and centrifuged at 7500 *g* for 5 min at 4 °C. Finally, the air-dried pellet was re-dissolved in RNase-free water. The quantity and quality of the extracted RNA were measured with E-Spect (Malcon, Japan). In total, 200 ng of total RNA was reverse-transcribed with a PrimerScriptTM RT Kit (TaKaRa) for mRNA while quantitative real-time PCR for mRNA was performed in a 96-well plate using a SYBR Premix Ex Taq Real Time PCR Kit (TaKaRa). Amplification reactions were carried out in a final volume of 20 μL and were performed on an Mx3005P Stratagene under the following thermal cycling conditions: (denaturation at 95 °C for 30 s [1×], followed by 40 cycles of denaturation [95 °C, 30 s], annealing [60 °C, 10 s] and extension [72 °C, 15 s]). Rat *Gapdh* was used as an endogenous reference. The primers used for detecting *Phgdh* and *Gapdh* expression are shown in Table 1. The comparative cycle threshold method was performed for relative quantification. The sequences of the primers were as follows: PHGDH forward, 5′- GATGAAAGATGGCAAATGGGA -3′; PHGDH reverse, 5′- GCGGGGTATGGACAGTGATG -3′. GAPDH forward, 5′-TGGAGTCTAGGCGTCTT -3′; GAPDH reverse5′- TGTCATATTTCTCGTGGTTCA − 3′.

**Table 1:** The primers used for the real-time PCR

Gene	Forward Primer (5-3)	Reverse Primer (5-3)
GAPDH	TGGAGTCTACTGGCGTCTT	TGTCATATTTCTCGTGGTTCA
PHGDH	GATGAAAGATGGCAAATGGGA	GCGGGGTATGGACAGTGATG

### Cell culture

Sertoli cells (a mouse testis Sertoli cell line) were purchased from American Type Culture Collection (ATCC, Manassas, VA, USA) and cultured in DMEM with 10% FBS at 37 °C in an incubator with an atmosphere of 5% CO2. For the experiments, sertoli cells were adherently cultured in 100-mm tissue culture dishes and reached 60 ~ 70% density before use.

### Small interfering RNA (siRNA) and transient transfection

PHGDH siRNA was used to silence the PHGDH gene. A scrambled sequence siRNA (siNCtrl) was used as a negative control. The siRNA transfection was optimized using Lipofectamine2000-RNAimax (Invitrogen, Carlsbad, CA, USA), according to the manufacturer s instructions. Briefly, siRNA and lipofectamine were diluted separately in Opti-MEM (Gibco) and incubated at room temperature for 5 min. Then, the two solutions were gently mixed and incubated for 15 min. Finally, the mixture was added to plated cells, and after 48 h, the cells were analyzed using the following assays.

### Flow cytometry for cell apoptosis and cell cycle

PHGDH siRNA and a negative control siRNA were transfected as mentioned above.For cell apoptosis analysis, cells were prepared with the PE Annexin V Apoptosis Detection Kit I (BD Biosciences, New Jersey, USA) according to the manufacturer’s recommendations. For cell cycle analysis, cells were fixed and permeabilized by 75% ethanol, and were stained by PI/RNase Staining Buffer (BD Biosciences, New Jersey, USA) after incubation at − 20 °C overnight. The cell apoptosis ratio and cell cycle profile were detected by FAC Station (FV500,Beckman Coulter, Brea, USA), and the raw data were analyzed using FlowJo 10.0.7 software (FlowJo, Oregon, USA).

### Western blot analysis

Total cells washed twice with cold PBS and lysed with RIPA (lysis buffer radioimmunoprecipitation assay) (Beyotime Institute of Biotechnology, China) and PMSF (protease inhibitors phenylmethanesulfonyl fluoride) (Beyotime Institute of Biotechnology, China). The protein concentration was determined by utilizing bicinchoninic acid (BCA) protein assay kit (P0010S, Beyotime Biotech, China). Equal amounts of protein (~ 10 μg) were separated by 10% sodium dodecyl sulfate polyacrylamide gel electrophoresis (SDS-PAGE) and then transferred to 0.45 μm polyvinylidine difluoride filter (PVDF) membranes. The membranes were blocked with 5% BSA for 1 h and then the membranes were incubated at 4 °C overnight with primary antibodies. Next, the membranes were washed three times with TBST for at least 15 min, and probed with HRP linked secondary antibodies for 1 h at room temperature. Protein bands were visualized employing the enhanced chemiluminescence detection kit (Thermo scientific, USA). The gray value of each protein band was analyzed by Image Lab software.

The primary antibodies were: PHGDH,PKM-2,PSPH and β-actin. All these antibodies were purchased from Abcam.

### Glucose consumption and lactate production

PHGDH siRNA and a negative control siRNA were transfected as mentioned above.After 48 h, the media were collected for measurement of glucose and lactate concentrations as determined by glucose (GO) assay kit (Sigma) and lactate assay kit (Biovision). Glucose consumption and lactate production were normalized by cell numbers.

### Measurement of LDH activities

PHGDH siRNA and a negative control siRNA were transfected as mentioned above. After 48 h, the media were collected for measurement of LDH activities as determined by colorimetric assay kits in accordance with the manufacturer protocols.

### Protein-protein interaction (PPI) network construction

Using the Search Tool for the Retrieval of Interacting Genes (STRING) database (http://www.string-db.org/), a PPI network related to PHGDH, PSPH, PSAT1 and PKM2 was established. The interactions procured included known interactions and predicted interactions.

### Statistical analysis

Data were statistically analyzed using GraphPad Prism 8.0 software, and were presented as the mean ± standard deviation (SD). An unpaired t-test was used to analyze the differences between the two groups. One-way ANOVA was used to analyze the intergroup differences among multiple groups. *P* < 0.05 was considered statistically significant.

## Results

### The lactate on the varicocele side of the patients decreased

**Fig. 1:**
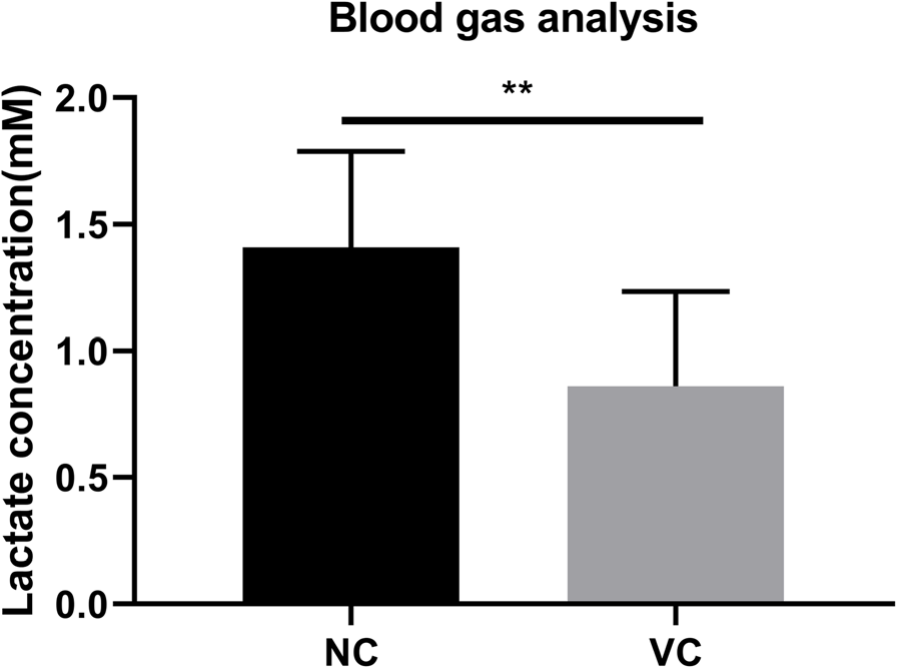
The lactate on the affected side of the varicocele decreased. Peripheral venous blood and affected side venous blood of varicocele patients were extracted respectively for blood gas analysis to detect lactate concentration. *N* = 10; *P < 0.05,**P < 0.01; Student’s t-test was used

The blood gas analysis results showed that compared with the peripheral venous blood, the lactate concentration in the varicocele side of the patients decreased significantly (Fig. 1).

### Testicular protein PHGDH was down-regulated in varicocele

**Fig. 2:**
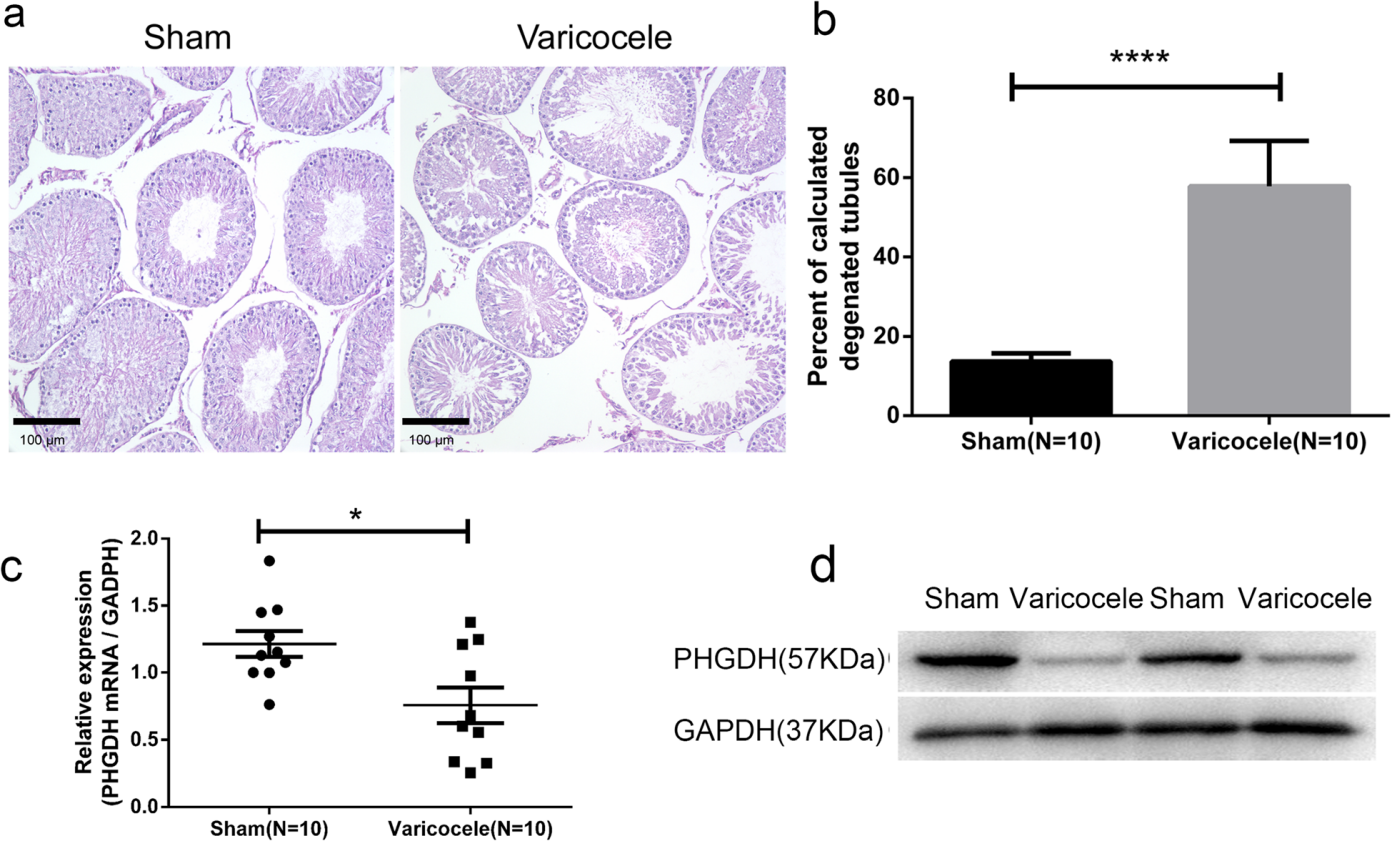
Testicular protein PHGDH was down-regulated in varicocele patients and rat varicocele model. **a** Hematoxylin and eosin stained testicular tissues in 8-weeks experimental varicocele rats, the varicocele rats showed more degeneration of the seminiferous tubules. **b** The percentages of degenerating seminiferous tubules in the varicocele group were significantly increased compared to the sham group. **c** The PHGDH mRNA expression was determined by quantitative Real-Time PCR, rat GAPDH was used as an endogenous reference. **d** The PHGDH protein expression was determined by western blot, rat GAPDH was used as an endogenous reference. *N* = 5; *P < 0.05,****P < 0.0001; Student’s t-test was used

PHGDH was of interest because of its involvement in lactic acid metabolism, and we chose to test our hypothesis in a rat model. Eight weeks after initial surgery, the mean diameter of the LSV in the varicocele group was significantly larger than that of the sham group (1.58 ± 0.05 mm versus 0.24 ± 0.01 mm, respectively, *P* < 0.0001, Supplementary Fig. 1). Histological assessment of the left testes showed that the proportion (%) of degenerating seminiferous tubules was significantly higher in the varicocele group than in the sham group (57.83 ± 3.63% versus 13.73 ± 0.65%, respectively, *P* < 0.0001, Fig. 2a, b), which indicated clear impairment of spermatogenesis. The mRNA and protein expression levels of PHGDH were examined with qRT-PCR and Western blotting, respectively, in the left testes from the two groups of rats. Notably, PHGDH mRNA and protein expression was obviously reduced in the left testes from the varicocele group relative to the sham group (Fig. 2c, d).

### PHGDH knockdown inhibited sertoli cells glycolysis and lactate production

**Fig. 3:**
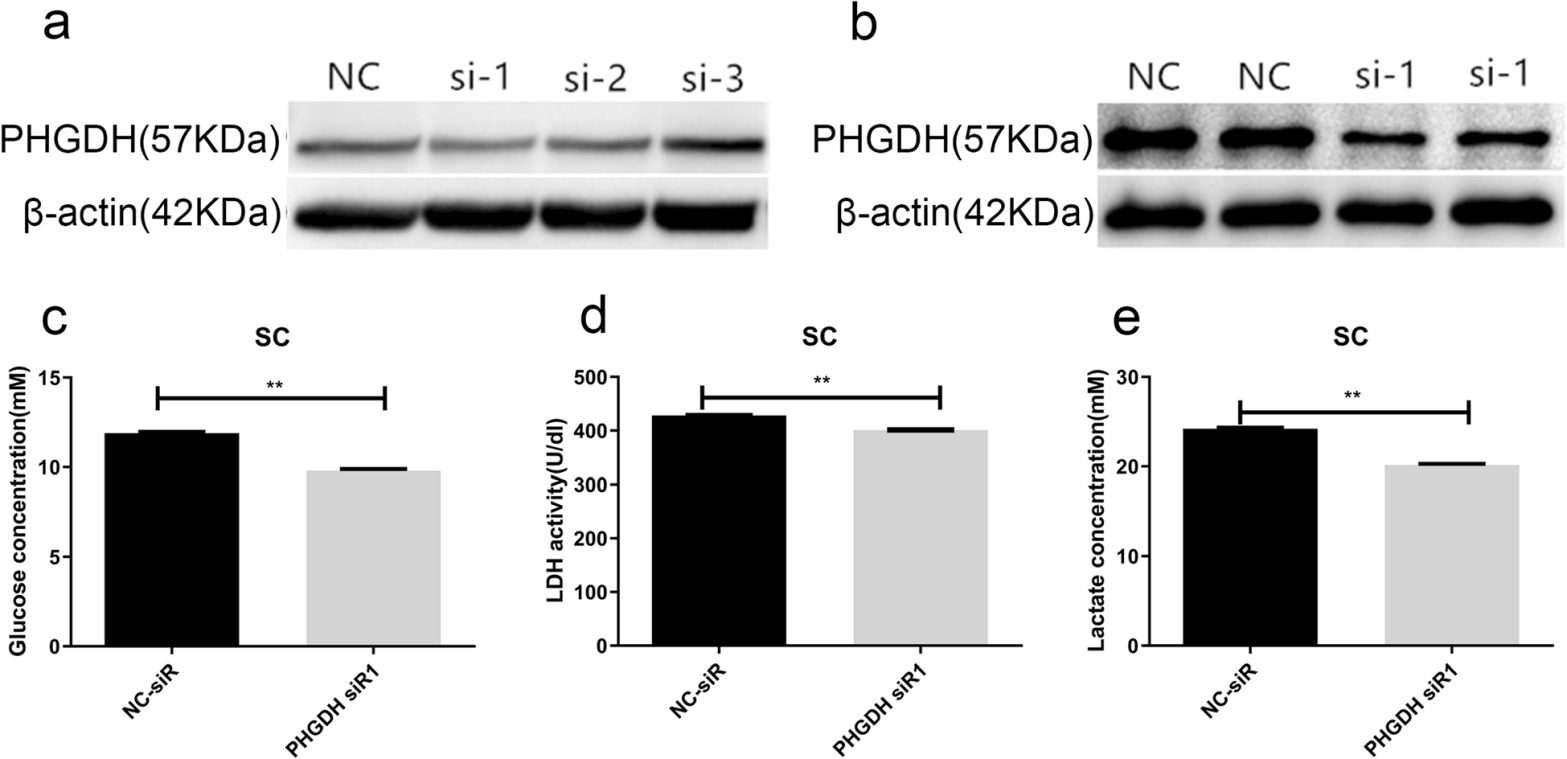
PHGDH knockdown inhibited sertoli cells aerobic glycolysis and lactate production. **a**, **b** PHGDH knockdown efficiency at protein level was detected by Western blot. **c**, **d** After transiently transfection sertoli cells with NC siR or PHGDH siR1 for 48 h, the media were then collected for analysis of glucose consumption (**c**) and LDH activities (**d**). **e** The lactate production of sertoli cells determined by lactate assay kit. N = 5; *P < 0.05,**P < 0.01; Student’s t-test was used

In order to investigate the role of PHGDH in sertoli cells, the expression of PHGDH was knocked down by PHGDH siRNA. sertoli cells were transfected with a scrambled sequence siRNA (siNCtrl) and three PHGDH siRNA. the protein level of PHGDH were inhibited significantly by PHGDH siR1 (Fig. 3a, b). Because the production of lactate to fulfill the energy needs of spermatogenesis is a crucial function of sertoli cells, and PHGDH may cause changes in cell lactate production by affecting glycolysis, we wondered whether PHGDH Knockdown regulate sertoli cells glycolysis. After transiently transfection sertoli cells with NC siR or PHGDH siR1 for 48 h, PHGDH knockdown significantly decreased the glucose consumption (Fig. 3c) and the LDH activities (Fig. 3d) of sertoli cells. We further examined the lactate production of sertoli cells,which showed that PHGDH knockdown inhibited the lactate production of sertoli cells (Fig. 3e). These results indicate that PHGDH knockdown inhibited the glycolysis of sertoli cells and resulted in the reduction of lactate production.

### PHGDH regulates the expression of PSPH and PKM2 expression in sertoli cells

**Fig. 4:**
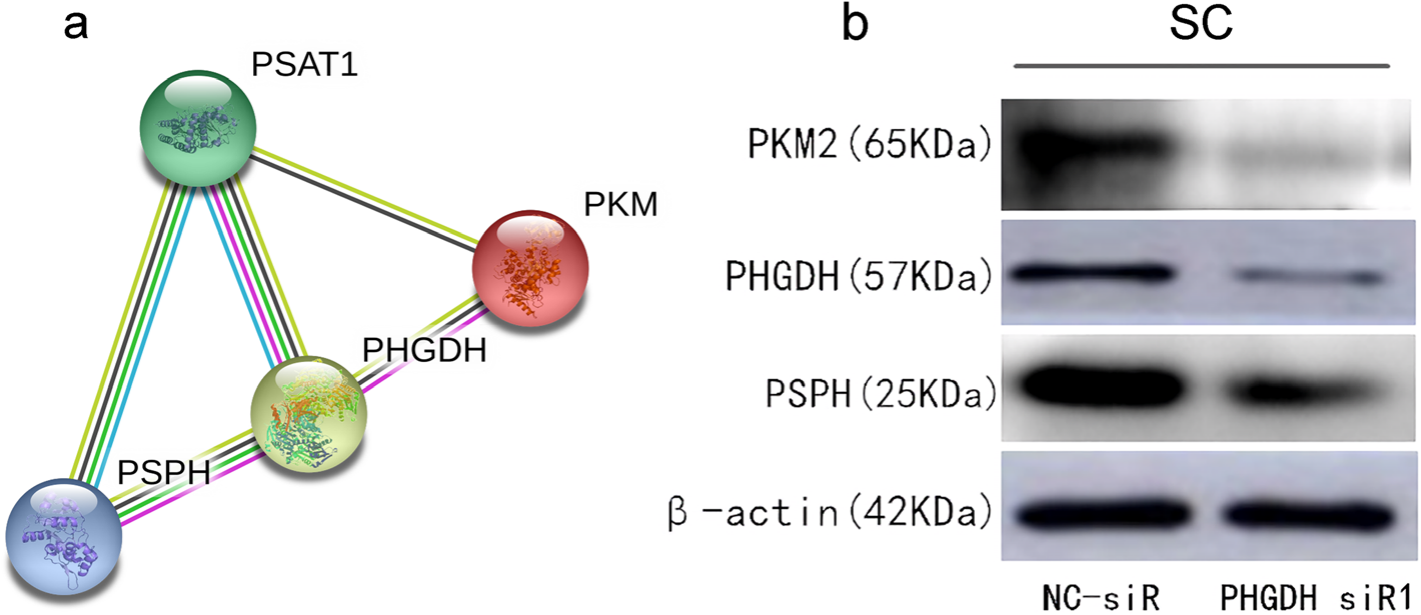
PHGDH regulates the expression of PSPH and PKM2 expression in sertoli cells. **a** PPI network showed that PSPH, PSAT1 and PKM2 were indeed correlated with PHGDH. **b** The protein expression levels of PSPH and PKM2 in PHGDH-siRNA transfected cells and empty vector-transfected cells were examined by western blot analysis, Western blot experiment was repeated three separate times

To investigate the mechanism how PHGDH regulates sertoli cell glycolysis and lactate production, we constructed a PPI network using the STRING database, It was verified that PSPH, PSAT1 and PKM2 were indeed related to PHGDH (Fig. 4a). We performed western blot to access the effects of PHGDH on the expression of PSPH and PKM2. Our results showed that knockdown of PHGDH remarkably decreased the expression of PSPH and PKM2 (Fig. 4b).

### PHGDH knockdown inhibited sertoli cells growth

**Fig. 5:**
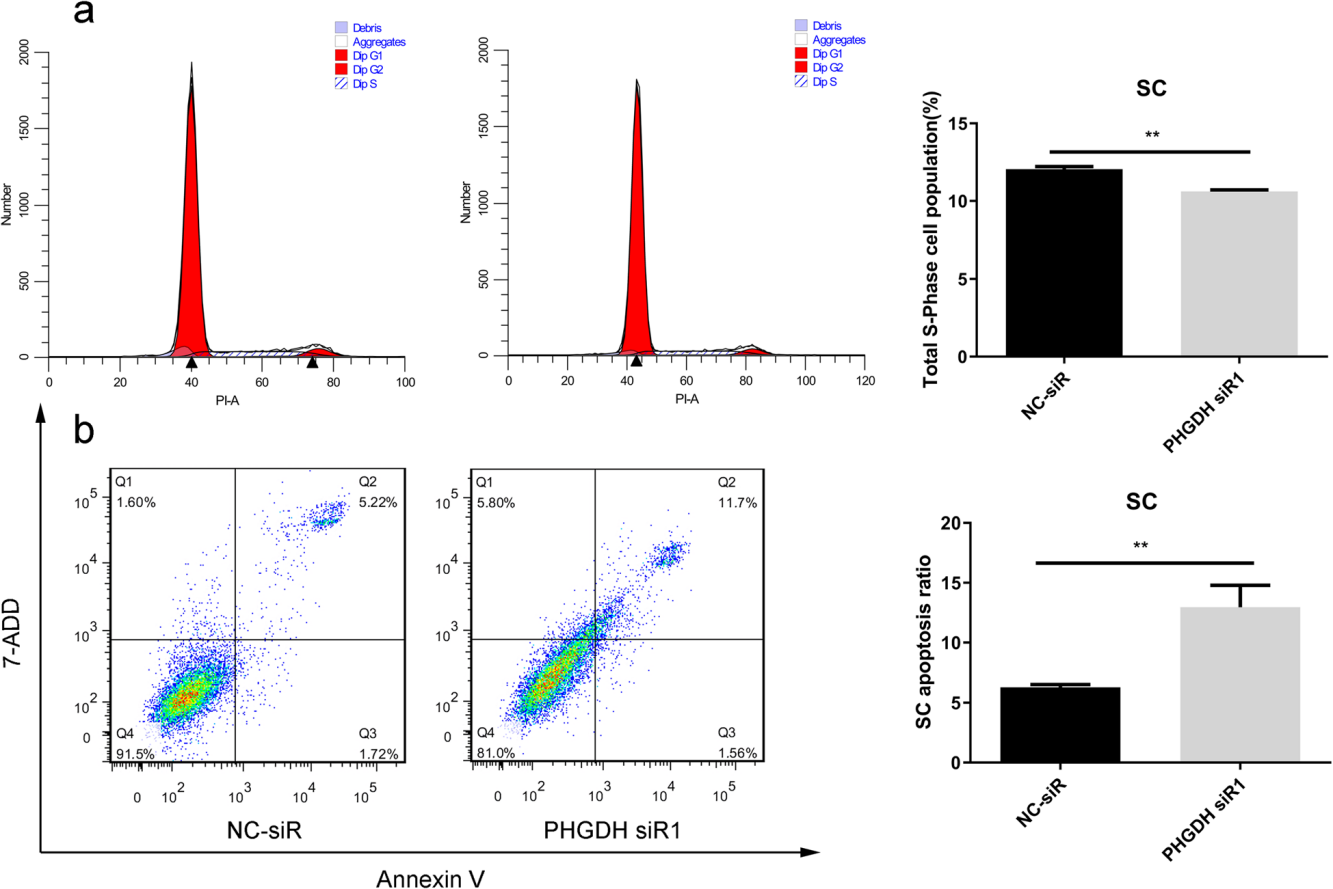
PHGDH knockdown inhibited sertoli cells growth. **a**, **b** Flow cytometry for cell cycle (**a**) and apoptosis (**b**) [apoptosis ratio was calculated as (Q2 + Q3)/(Q1 + Q2 + Q3 + Q4)] after PHGDH knockdown in sertoli cells. N = 5; *P < 0.05,**P < 0.01; Student’s t-test was used

After transiently transfection sertoli cells with NC siR or PHGDH siR1 for 48 h, cell cycle and cell apoptosis assays indicated that PHGDH knockdown decreased the proportion of cells in S-phase (Fig. 5a) and increased the percentage of apoptotic cells (Fig. 5b) in sertoli cells compared with control groups. These results indicate that PHGDH might play a key role in sertoli cells growth.

## Discussion

Although varicocele is considered to be one of the leading causes of male infertility [14], the precise mechanism underlying how varicocele leads to male infertility is not completely understood. As early as 1981, a study found that the production of lactate in the varicocele side of the patients was decreased, which was speculated to be caused by the absence of glycolysis [15]. Another study detected a decrease in the level of lactate in spermatic plasma in infertile patients with varicocele [16]. Using the varicocele model of rabbit, it was found that the single spermatic vein could cause the decrease of lactate and pyruvate in both spermatic veins [17]. In the above studies, it was found that varicocele could cause the decrease of lactate in the varicocele side and the seminal plasma. In our study, we collected the blood in the varicocele side and the peripheral venous blood of the patients for blood gas analysis, found that the lactate on the varicocele side of the patients showed a decrease. In the testicles, lactate is mainly generated by sertoli cells through the glycolysis pathway, which supplies energy to spermatogonial cells and maintains their proliferation and differentiation [18]. The reduction of lactate will affect spermatogenesis [8]. Therefore, this clinical phenomenon has aroused our interest.

To investigate the mechanism behind the reduction of lactate, we collected a small amount of testicular tissue from patients with varicocele and found that the glycolysis related proteins PHGDH was down-regulated in varicocele patients (Supplementary File1). Due to the small sample size, we chose to verify the results in experimental rat varicocele model. Clearly, the current surgically-induced varicocele rat model differs from the situation seen in clinical patients with varicocele in several respects, such as venous anatomy, and the duration of varicocele. However, the rat model is widely accepted and used for investigating the pathophysiology of varicocele [19]. Finally, we found that testicular protein PHGDH was down-regulated in varicocele patients and rat varicocele model, the decrease of PHGDH expression may be the reason for the decrease of lactate.

PHGDH, encoding 3-phosphoglycerate dehydrogenase, is located on chromosome 1p12 and is widely distributed in organisms and in tissues [20]. PHGDH is the frst enzyme branching from glycolysis in a three-step serine biosynthetic pathway uses NAD+ as a cofactor to oxidize 3-phosphoglycerate into phosphohydroxypyruvate. The product is then subsequently converted into phosphoserine via transamination by phosphoserine aminotransferase (PSAT1) and, ultimately, to serine via phosphate ester hydrolysis and the enzyme PSPH, serine is also an activator of PKM2 in the glycolysis. Over recent years, an increasing number of studies have focused upon PHGDH in cancer research, which is found to exhibit elevation and controlled flux throughout the serine biosynthetic pathway in cancer cells [21]. Interestingly, The overexpression of PHGDH is often associated with progression of cancers, and the inhibitors of PHGDH reduce the glycolysis and suppress the growth of cancers [22]. PHGDH amplification may alter glucose metabolism in human melanoma, and may result in changes in redox status, energy metabolism, and potentially, other signaling functions [10]. These observations suggested that PHGDH was implicated in cell growth, proliferation and glycolysis. PHGDH mRNA was found to be expressed at high levels in testicular tissue [20], and PHGDH was considered as testicular spermatogenesis-related panels [23]. PHGDH was also shown to be major antigen for ovarian autoimmunity associated with female infertility [24]. However, the associated investigation in the reproductive system remains limited.

In order to investigate the role of PHGDH in sertoli cells, we constructed a sertoli cell of PHGDH down-regulation model. Down-regulation of PHGDH in sertoli cells significantly decreased the glucose consumption, LDH activities and lactate production in the sertoli cells, indicating that the low expression of PHGDH ultimately led to a decrease in lactate production by affecting the glycolysis. Western blot was conducted to investigate the effects of PHGDH on the expression of PSPH and PKM2, and the results showed that the down-regulation of PHGDH significantly reduced the expression of pathway protein PSPH and PKM2, leading to the reduction of lactate production.

Autophagy is considered to be a process conserved during evolution that plays an important role in physiological and pathological conditions. Its main role is the degradation of harmful cytoplasmic components such as damaged organelles and poorly folded proteins that are no longer needed. In different diseases, the relationship between autophagy and apoptosis is not the same [25]. Study found that autophagy can aggravate the apoptosis of malignant glioma cells [26]. Another study found that autophagy could improve the ability of cells to fight infection, which reduces the apoptosis of infected cells [27]. And previous studies have shown that in varicocele, the decrease of glycolysis products is an important cause of germ cell autophagy, and long-term autophagy increases germ cell apoptosis [5]. Our results showed that PHGDH knockdown can promote apoptosis and inhibit cell cycle to affect sertoli cell growth. In fact, many previous studies have shown that varicocele activates apoptosis in seminiferous epithelial cells leading to sertoli-mediated phagocytosis of apoptotic germ cells [28]. These studies suggests that autophagy induced by long-term hypoxia in varicocele is an important cause of apoptosis [29]. Our study suggests that the decrease in glycolysis products due to low expression of PHGDH may also be an important cause of apoptosis in varicocele. It may also lead to abnormal testicular energy metabolism in patients with varicocele, and ultimately affect spermatogenesis and lead to infertility. This may help to better understand the major proteins contributing to male infertility in varicocele, further exploring the mechanisms underlying male infertility, and developing novel treatments for varicocele.

## Conclusions

In summary, our current findings indicate that varicocele lead to the low expression of PHGDH in sertoli cells and the low expression of PHGDH ultimately led to a decrease in lactate production by affecting the glycolysis pathway. Moreover, PHGDH knockdown can promote apoptosis and inhibit cell cycle to affect cell growth. That may be one of the important causes of impairment of spermatogenesis and male infertility. Nevertheless, there are some limitations to this study. First, we discovered that testicular protein PHGDH was down-regulated in varicocele male adults with asthenospermia (Supplementary File1), due to the small sample size, we chose to verify the results in experimental rat varicocele model. Second, we did not further investigate the effect of lactate reduction on the proliferation and differentiation of spermatogonia, and is therefore under progress in our lab.

At present, the literature relating to PHGDH in spermatogenesis is quite limited and many questions, particularly related to mechanisms, still remain to be elucidated regarding the mechanisms underlying varicocele-related infertility. Our study provides a new viewpoint to reveal the mechanisms underlying metabolism-associated male infertility. Moreover, these data highlight that PHGDH is a potential modulatory biomarker in varicocele, which may help in the development of a new therapeutic target for male infertility.

## Supplementary information

**Additional file 1.**

**Additional file 2.**

**Additional file 3.**

## Data Availability

The dataset supporting the conclusions of this article is included within the article.

## Abbreviations

PHGDH: Phosphoglycerate dehydrogenase
PSPG: Phosphoserine phosphatase
PSAT1: Phosphoserine aminotransferase 1
PKM2: Pyruvate kinase M2
ANOVA: Analysis of variance
PPI: Protein-protein interaction
GO: Gene Ontology
iTRAQ: Isobaric tags for relative and absolute quantitation
