# Supplementary figures and images for: Down regulating PHGDH affects the lactate production of sertoli cells in varicocele
Source: Reprod Biol Endocrinol. 2020 Jul 14;18:70. doi: 10.1186/s12958-020-00625-9 (PMC7359552; doi:10.1186/s12958-020-00625-9)

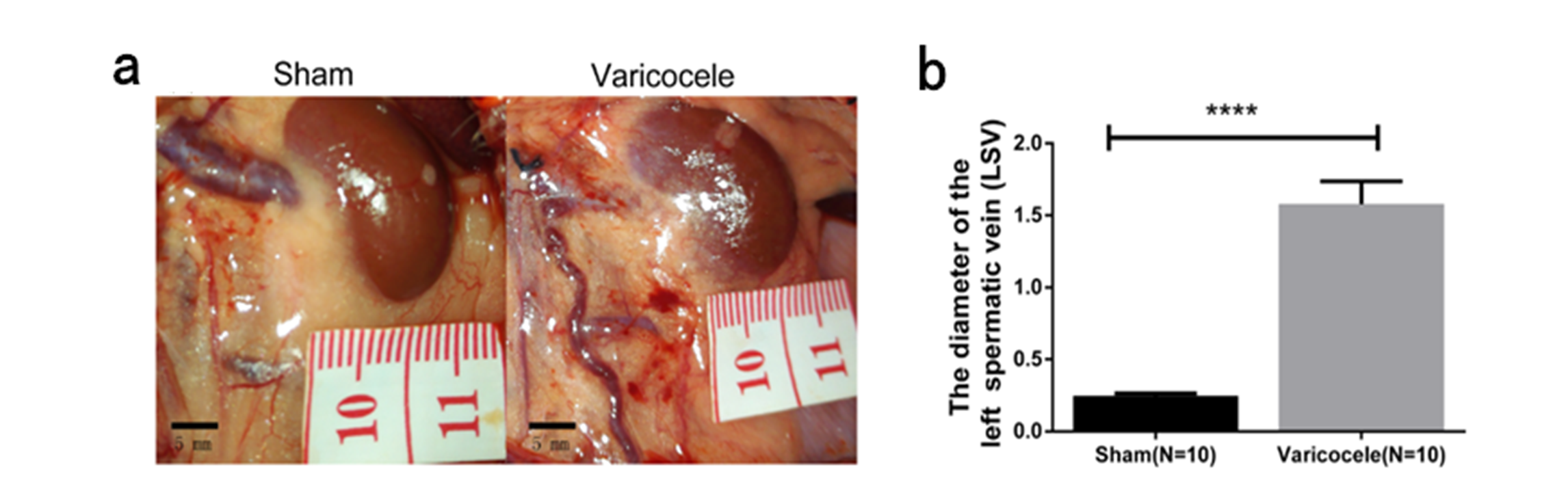

Supplement: Supplementary file 1 — Additional file 1. [file 12958_2020_625_MOESM1_ESM.tif]
