# Supplementary material for: Down regulating PHGDH affects the lactate production of sertoli cells in varicocele
Source: Reprod Biol Endocrinol. 2020 Jul 14;18:70. doi: 10.1186/s12958-020-00625-9 (PMC7359552; doi:10.1186/s12958-020-00625-9)
Supplement: Supplementary file 2 — Additional file 2. [file 12958_2020_625_MOESM2_ESM.doc]

**Table1.Down-regulation of 21 CDEs between the normal spermatogenesis and varicocele**

| **Protein Accession** | **Gene name** | **Protein description** | **MW [Da]** | **pI** | **AASC [%]** | **Score** | **S25/S1 Ratio** | **overall trend** |
| --- | --- | --- | --- | --- | --- | --- | --- | --- |
| P01876 | IGHA1 | Ig alpha-1 chain C region | 42745 | 6.08 | 38 | 245 | 0.436 | down |
| P02763 | ORM1 | Alpha-1-acid glycoprotein 1 | 28288 | 4.93 | 24.9 | 312 | 0.581 | down |
| Q99584 | S100A13 | Protein S100-A13 | 15723 | 5.91 | 40.8 | 96 | 0.653 | down |
| P80404 | ABAT | 4-aminobutyrate aminotransferase, mitochondrial | 66517 | 8.17 | 29.2 | 197 | 0.651 | down |
| Q02539 | HIST1H1A | Histone H1.1 | 39473 | 10.99 | 22.8 | 316 | 0.503 | down |
| Q08380 | LGALS3BP | Galectin-3-binding protein | 72286 | 5.13 | 19.8 | 432 | 0.651 | down |
| P04196 | HRG | Histidine-rich glycoprotein | 67507 | 7.09 | 13.9 | 242 | 0.743 | down |
| P02765 | AHSG | Alpha-2-HS-glycoprotein | 45270 | 5.43 | 13.9 | 229 | 0.617 | down |
| P01859 | IGHG2 | Ig gamma-2 chain C region | 44110 | 7.66 | 29.1 | 402 | 0.612 | down |
| P06727 | APOA4 | Apolipoprotein A-IV | 54193 | 5.28 | 24 | 106 | 0.676 | down |
| A6NGU5 | GGT3P | Putative gamma-glutamyltranspeptidase 3 | 68611 | 6.68 | 4.4 | 86 | 0.724 | down |
| P04217 | A1BG | Alpha-1B-glycoprotein | 58440 | 5.56 | 21.2 | 227 | 0.64 | down |
| P01019 | AGT | Angiotensinogen | 60098 | 5.87 | 13 | 225 | 0.667 | down |
| P22352 | GPX3 | Glutathione peroxidase 3 | 30632 | 8.26 | 28.8 | 99 | 0.727 | down |
| P36955 | SERPINF1 | Pigment epithelium-derived factor | 54972 | 5.97 | 28 | 218 | 0.736 | down |
| O43175 | PHGDH | D-3-phosphoglycerate dehydrogenase | 65569 | 6.29 | 34 | 313 | 0.728 | down |
| P00450 | CP | Ceruloplasmin | 143365 | 5.44 | 7.3 | 211 | 0.69 | down |
| Q9NZ08 | ERAP1 | Endoplasmic reticulum aminopeptidase 1 | 126293 | 6.02 | 10.6 | 62 | 0.741 | down |
| Q7Z2X7 | PAGE2 | P antigen family member 2 | 13300 | 4 | 79.3 | 116 | 0.686 | down |
| P78417 | GSTO1 | Glutathione S-transferase omega-1 | 35134 | 6.23 | 17 | 43 | 0.699 | down |
| P08311 | CTSG | Cathepsin G | 30682 | 11.19 | 23.1 | 133 | 0.776 | down |
