# Supplementary material for: Down regulating PHGDH affects the lactate production of sertoli cells in varicocele
Source: Reprod Biol Endocrinol. 2020 Jul 14;18:70. doi: 10.1186/s12958-020-00625-9 (PMC7359552; doi:10.1186/s12958-020-00625-9)
Supplement: Supplementary file 3 — Additional file 3. [file 12958_2020_625_MOESM3_ESM.docx]

| Samples | Semen volume  (ml) | Total sperm number (million) | Sperm concentration  (million/ml) | Vitality  (%) | Progressive motility  (%) |
| --- | --- | --- | --- | --- | --- |
| 1 | 4.0 | 318.7 | 79.7 | 40.8 | 27.4 |
| 2  3  4  5  6  7  8  9  10 | 3.0  5.0  3.0  4.0  3.0  4.5  5.0  5.0  4.0 | 84.9  203.9  15.5  145.6  107.0  304.8  375.2  315.8  588.3 | 28.3  40.8  5.2  36.4  35.7  67.7  75.0  63.2  147.1 | 0  19.9  31.4  28.0  41.8  48.1  46.3  43.2  45.6 | 0  8.4  12.2  21.1  30.8  31.7  36.9  35.8  37.2 |

Table1: Semen parameters in patients with varicocele.
